# Significance of Dermoscopy in Association with Clinical Features in Differentiation of Basal Cell Carcinoma and Benign Trichoblastic Tumours

**DOI:** 10.3390/cancers14163964

**Published:** 2022-08-17

**Authors:** Martyna Sławińska, Anna Płaszczyńska, Joanna Lakomy, Krzysztof Pastuszak, Wojciech Biernat, Monika Sikorska, Roman J. Nowicki, Michał Sobjanek

**Affiliations:** 1 Department of Dermatology, Venereology and Allergology, Faculty of Medicine, Medical University of Gdańsk, 80-210 Gdańsk, Poland; 2 Department of Pathomorphology, Faculty of Medicine, Medical University of Gdańsk, 80-210 Gdańsk, Poland; 3 Department of Algorithms and System Modeling, Faculty of Electronics, Telecommunication and Informatics, Gdansk University of Technology, 80-233 Gdańsk, Poland; 4 Laboratory of Translational Oncology, Intercollegiate Faculty of Biotechnology, Medical University of Gdańsk, 80-210 Gdańsk, Poland; 5 Centre of Biostatistics and Bioinformatics Analysis, Medical University of Gdansk, 80-210 Gdańsk, Poland

**Keywords:** dermoscopy, dermatoscopy, basal cell carcinoma, trichoblastic tumour, trichoepithelioma, trichoblastoma, desmoplastic trichoepithelioma

## Abstract

**Simple Summary:**

Although basal cell carcinoma (BCC) can, in the majority of cases, be diagnosed based on clinical and dermoscopic assessment, a potential overlap with benign adnexal skin tumours seems to exist, including trichoblastic tumours (TT). The aim of this study was to analyse clinical and dermoscopic features of benign TT and BCC to develop a diagnostic algorithm with a potential utility in clinical practice. Despite differences in frequency of clinical and dermoscopic features between BCC and TT in the studied group, differential diagnosis based on these variables is not reliable. Histopathological examination remains a diagnostic gold standard in differentiation of BCC and TT.

**Abstract:**

**Background**: Although basal cell carcinoma (BCC) can, in the majority of cases, be diagnosed based on clinical and dermoscopic assessment, a potential overlap with benign adnexal skin tumours seems to exist, including trichoblastic tumours (TT). **Methods:** Retrospective analysis of clinical and dermoscopic features of benign TT and BCC cases was performed to develop a diagnostic algorithm with a potential utility in clinical practice. **Results**: In the study, 502 histopathologically confirmed BCC cases were compared with 61 TT (including 44 TB (72.13%), 10 TE (16.39%) and 7 DTE (11.48%]). Patients in the BCC group were statistically older (mean age was 71.4 vs. 64.4 years, respectively; *p* = 0.009). BCC presented generally as larger tumours (mean tumour size 11.0 vs. 8.2 mm for the TT group; *p* = 0.001) and was more frequently associated with clinically visible ulceration (59.4% vs. 19.7%, respectively; *p* < 0.001). Comparison of lesion morphology, clinically visible pigmentation, and anatomical location did not show significant differences between the analysed groups. Dermoscopically visible ulceration was significantly more common in the BCC group compared to the TT group (52.2% vs. 14.8%; *p* < 0.0001). Pigmented structures, specifically brown dots and brown globules, were significantly more prevalent in the TT group (32.8% vs. 11.4%; *p* = 0.0001 and 29.5% vs. 8.2%; *p* <0.0001). Similarly, TT more commonly than BCC showed the presence of cloudy/starry milia-like cysts (26.2% vs. 11.6%; *p* = 0.0031) and yellow globules (16.4% vs. 7.2%; *p* = 0.033). **Conclusions:** Despite differences in frequency of clinical and dermoscopic features between BCC and TT in the studied group, differential diagnosis based on these variables is not reliable. Histopathological examination remains a diagnostic gold standard in differentiation of BCC and TT.

## 1. Introduction

Dermoscopic criteria for diagnosis of basal cell carcinoma (BCC) have been systematically evaluated and updated in recent years [1,2]. Although BCC can, in the majority of cases, be diagnosed based on clinical and dermoscopic assessment, a potential overlap with benign adnexal skin tumours seems to exist, including trichoblastic tumours [1]. This differential diagnosis should be considered especially in nodular, infiltrative, morpheaform, as well as mixed BCC variants (both pigmented and non-pigmented) [3]. To date, relatively few studies, with a limited number of patients, have explored dermoscopic features of trichoblastoma (TB), trichoepithelioma (TE), or desmoplastic trichoepithelioma (DTE) [4,5,6,7]. Trichoblastoma (TB) is a benign skin tumour with hair germ differentiation. Based on the growth pattern and configuration of mesenchymal and epithelial components, trichoblastomas (TBs) are grouped into seven types: small nodular, large nodular, retiform (giant solitary trichoepithelioma), cribriform (classic trichoepithelioma), racemiform (non-classic trichoepithelioma), columnar (desmoplastic trichoepithelioma), and adamantinoid (cutaneous lymphadenoma) [8]. In some cases, histopathological features of the mentioned subtypes overlap, thus reliable distinction may not be possible [9]. As mentioned, TE and DTE are currently considered as variants of TB.

Currently, the gold standard in differential diagnosis of the above-mentioned tumours and BCC is histopathological (and sometimes immunohistochemical) assessment. Differential diagnosis is important, as benign trichoblastic tumours differ in terms of growth character, planned surgical excision margin, and the risk of recurrence.

The aims of the study were to conduct detailed clinical and dermoscopic analysis of patients diagnosed with benign trichoblastic tumours, comparing them with a large cohort of patients with histopathologically confirmed BCC.

## 2. Materials and Methods

Medical records from a tertiary clinical centre from northern Poland (Department of Dermatology, Venerology and Allergology, Medical University of Gdańsk) including completely excised and histopathologically examined skin tumours between May 2016 and August 2021 were retrospectively analysed to identify all cases of BCC and benign TT (TB, TE, DTE). Sample biopsies, recurrent BCC, giant tumours (diameter > 50 mm), purely superficial BCC, as well as collision lesions were excluded from analysis. Additionally excluded was TB associated with Brook–Spiegler syndrome/multiple familial trichoepitheliomas and TB arising over sebaceous nevus. After quality assessment of clinical and dermoscopic pictures, 502 BCC cases and 61 TT were finally included in further analysis. All cases were retrieved from one image database, performed by the same videodermoscope (FotoFinder Vexia, Camera Medicam 800 HD) with the same methodology (non-polarised mode, with an ultrasound gel as immersion fluid, ×20 magnification) by dermoscopists working in a skin cancer unit, using dermoscopy in daily practice. All histopathological cases were assessed by two histopathologists experienced in dermatopathology (from Department of Pathomorphology, Medical University of Gdańsk). Clinical and dermoscopic pictures were independently analysed by predefined criteria by two investigators (M.Sł and M.So) blinded for histopathological diagnosis. In case of any discrepancy, a third investigator was consulted, and a decision was reached by consensus (M. Si). In addition to dermoscopic criteria, clinical data including age, gender, tumour location, tumour size, clinically visible pigmentation and ulceration, and lesion morphology (flat/elevated) were evaluated. As all pictures were performed using non-polarised mode, the structures visible only on polarised dermoscopy were not analysed.

### 2.1. Statistical Analysis

Data analysis was carried out using statistical software R (version 4.1.2, available on https://cran.r-project.org/), and κ statistic and percentage of positive concordance were calculated for interobserver agreement analysis of dermoscopic features. A *p* < 0.05 was considered statistically significant. Statistical tests used according to the analysed variables are mentioned in the Table 1 footnotes. If information was missing, no data imputation was used. Missing values were excluded from comparisons and statistical tests. The significance of the difference in the binary variables was analysed using the exact Fisher test. For the quantitative variables, the Shapiro–Wilk test was performed to evaluate whether the distribution is normal. A *t*-test was used to analyse the differences of variables with normal distribution. If the distribution significantly differed from normal, Mann–Whitney U test was used instead.

### 2.2. Diagnostic Algorithm Development

Concerning the number of patients in the study (relatively low for particular subtypes of trichoblastic tumours) and the control group, a binary classification algorithm was developed.

All patients with TT (TB, TE, DTE) were grouped together as benign changes. No data imputation was used; patients with missing data were excluded from the further analysis. The analysed cohort was split randomly into four subgroups while preserving the proportion of benign changes to BCC. Three of these groups were used to develop the algorithm. The fourth group was excluded from the process and was used only as the independent test set to evaluate the performance of the final model. To avoid information leak between the training set and the independent test set, results from the statistical analysis of the whole cohort of patients were not used in the development of the algorithm.

Three-fold cross-validation was used during the training. A classification algorithm was constructed using the XGBoost library [10]. Three initial models were developed, each using two folds as the training set, and the remaining fold as the validation set. For each of the models, ten variables with the highest positive impact on the accuracy of classification were selected. Accuracy was measured against the validation set. The final model was then prepared. Only variables that were selected in at least two of the initial models were considered. Based on the preceding analysis and clinical observations, the following variables were selected in the process of development of the final diagnostic algorithm: age, tumour size, presence of ulceration, and presence of the cloudy/starry milia-like cysts. All three folds were used for the training of the final model. The training process was repeated ten times, each time with a different random seed. The final algorithm managed to reach 79.23% AUC (95% CI: 69.69–88.76%) on the independent test set. Detailed ROC curves are included in the supplement. Feature importance was measured using the built-in XGBoost function which evaluates relative gain using the variable in the model.

## 3. Results

**Figure 1:**
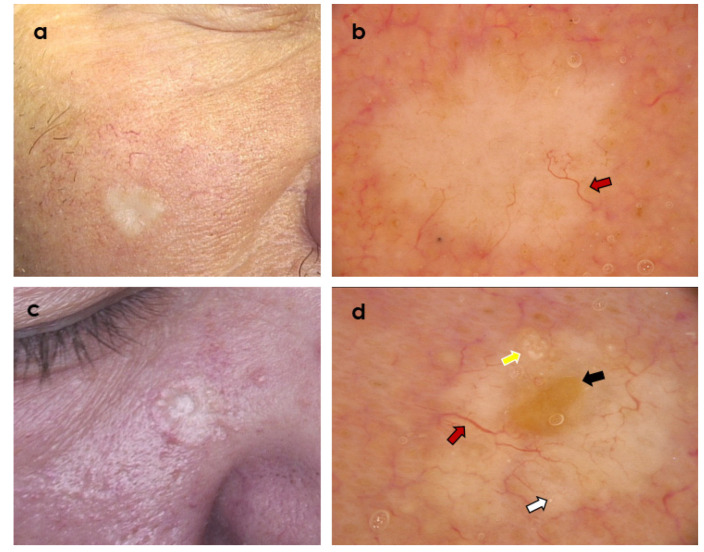
Two clinically similar tumours presenting as whitish plaque within the cheek area. (**a**,**b**) Desmoplastic trichoepithelioma; dermoscopy shows branched (arborizing) vessels (red arrow) over whitish background (Fotofinder Vexia; Camera Medicam 800 HD, ×20 magnification). (**c**,**d**) Morpheaform basal cell carcinoma; dermoscopy shows central ulceration (black arrow) and branched (arborizing) vessels (red arrow) over whitish background, starry milia-like cysts (white arrow) and Multiple Aggregated Yellow-White Globules (yellow arrow) (Fotofinder Vexia; Camera Medicam 800 HD, ×20 magnification).

**Figure 2:**
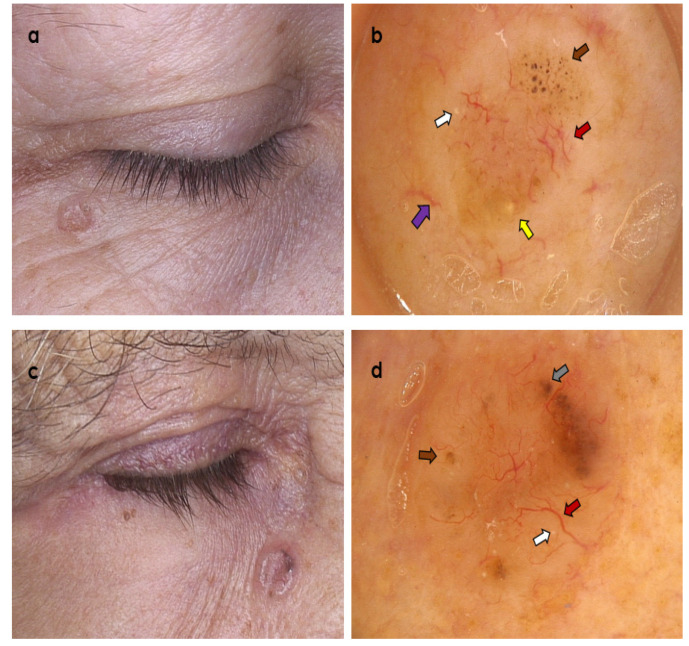
Two clinically similar tumours presenting as non-ulcerated, partially pigmented nodules within the lower eyelid region. (**a**,**b**) Trichoblastoma; dermoscopy shows branched (arborizing) vessels over whitish background (red arrow), superficial fine telangiectasia (violet arrow), brown dots and globules (brown arrow), yellow globules (yellow arrow), and starry milia-like cysts (white arrow) (Fotofinder Vexia; Camera Medicam 800 HD, ×20 magnification). (**c**,**d**) Nodular basal cell carcinoma; dermoscopy shows branched (arborizing) vessels (red arrow), blue-grey globules (grey arrow), brown dots and globules (brown arrow), starry milia-like cysts (white arrow) (Fotofinder Vexia; Camera Medicam 800 HD, ×20 magnification).

**Figure 3:**
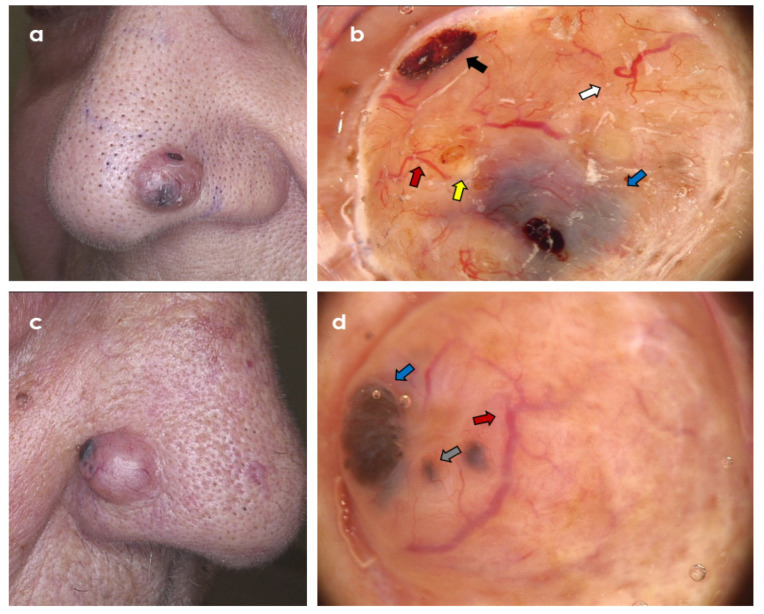
Two clinically similar, partially pigmented tumours within the ala nasi. (**a**,**b**) Trichoblastoma; dermoscopy shows branched (arborizing) vessels (red arrow), ulcerations (black arrow), large blue ovoid nests (blue arrow), yellow globules (yellow arrow), and starry milia-like cysts (white arrow) (Fotofinder Vexia; Camera Medicam 800 HD, ×20 magnification). (**c**,**d**) Nodulo-infiltrative basal cell carcinoma; dermoscopy shows branched (arborizing) vessels (red arrow), blue-grey globules (grey arrow), and large blue ovoid nest (blue arrow) (Fotofinder Vexia; Camera Medicam 800 HD, ×20 magnification).

**Figure 4:**
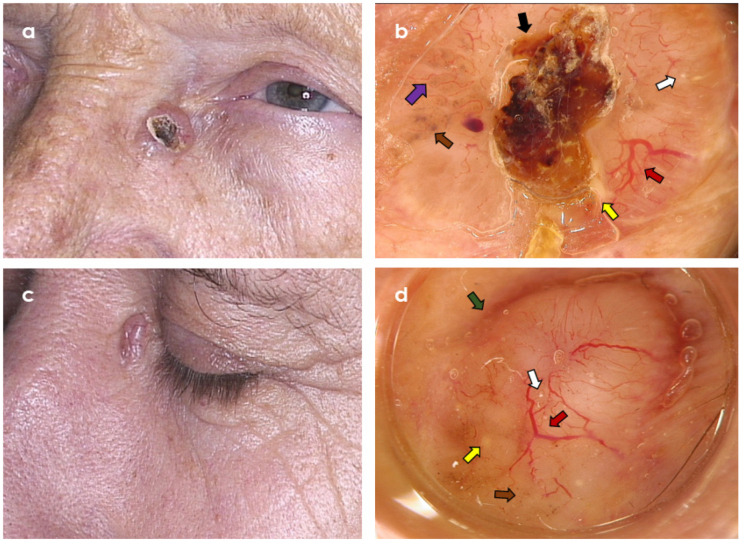
Two clinically similar tumours in the area of left medial eye canthus. (**a**,**b**) Trichoepithelioma; dermoscopy shows branched (arborizing) vessels (red arrow), superficial fine telangiectasia (violet arrow), central ulceration (black arrow), brown dots and globules (brown arrow), yellow globules (yellow arrow), and starry milia-like cysts (white arrow) (Fotofinder Vexia; Camera Medicam 800 HD, ×20 magnification). (**c**,**d**) Nodular basal cell carcinoma; dermoscopy shows branched (arborizing) vessels (red arrow), brown dots (brown arrow), starry milia-like cysts (white arrow), yellow globules (yellow arrow), and peripheral brown structureless areas (green arrow) (Fotofinder Vexia; Camera Medicam 800 HD, ×20 magnification).

**Figure 5:**
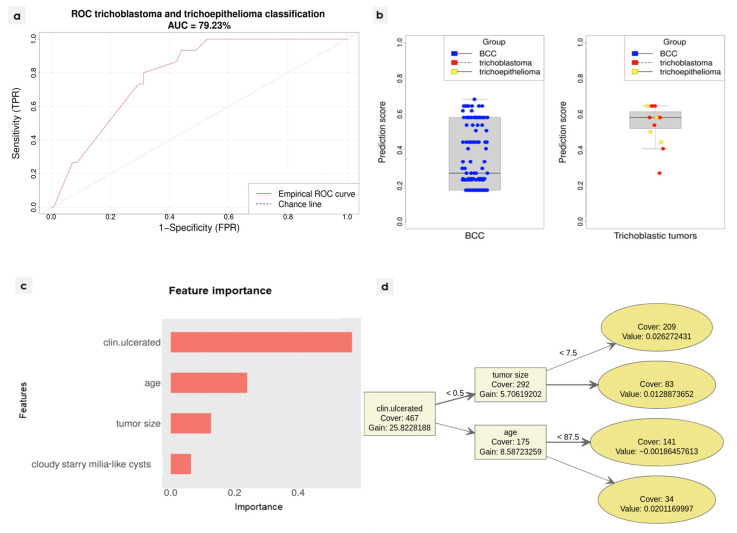
(**a**) ROC curve of the classification algorithm tested against the independent test set; (**b**) prediction scores for each sample from the independent test set. The scores may be interpreted as the similarity of the particular sample with the samples from the training set. The final classification would depend on the assumed cut-off level. The closer the score is to 0, the more the sample resembles basal cell carcinoma (BCC). On the left, samples for which the real diagnosis is BCC. On the right, samples with trichoblastic tumours (TT). (**c**) Feature importance of the final classifier, as measured on the training set using built-in XGBoost method. (**d**) The final algorithm tree. The result is a number in (0.1) range which is later transformed into binary decision based on the determined cut-off. Please note that the features were coded as numeric variables, hence “>0.5” in case of binary features actually means the presence of the feature.

The summary of the analysed clinical and dermoscopic variables is presented in Table 1 and Figure 1, Figure 2, Figure 3, Figure 4 and Figure 5.

**Table 1:** Clinical and dermoscopic features in the studied groups—basal cell carcinoma vs. trichoblastic tumours.

Clinical/Dermoscopic Variable	BCC *n* = 502	Trichoblastic Tumours *n* = 61	*p*	Concordance between Both Readers: Positive Agreement, %/Presence According to Both Readers, %	Concordance between Both Readers: Cohen Kappa (95% CI) ^1^
**Clinical variables**					
Mean (years)	71.4	64.4		-	
Median (years) (age range)	73 (12–95)	68 (9–94)	**0.0152** ^3^	-	
Females/males	261 F/241 M (52.0% F)	27 F/34 M (44.3% F)	0.279 ^2^	-	
Tumour size—mean (median) (mm) ^4^	10.998 (9)	8.197 (7)	**0.001** ^3^	-	
Tumour morphology—flat	96 (19.12%)	9 (14.75%)	0.1263 ^2^	-	
Tumour morphology—elevated	406 (80.88%)	52 (85.25 %)	0.1263 ^2^	-	
Clinically visible tumour ulceration	298 (59.4%)	12 (19.7%)	**<0.001** ^2^	-	
Non-pigmented tumour	437 (87.05%)	47 (77.05%)	0.0675 ^2^	98.97–99.11%	0.964 (0.933–0.995)
Pigmented structures within <25% tumour surface	19 (3.78%)	2 (3.28%)	0.6888 ^2^	72–98.76%	0.831 (0.708–0.954)
Pigmented structures within 25–50% tumour surface	27 (5.38%)	6 (9.84%)	0.2544 ^2^	68.97–98.4%	0.808 (0.685–0.93)
Pigmented structures within >50% tumour surface	27 (5.38%)	6 (9.84%)	0.2544 ^2^	86.84–99.11%	0.925 (0.859–0.99)
Location—scalp	21 (4.18%)	1 (1.64%)	0.4957 ^2^	-	
Location—face	384 (76.49%)	48 (78.69%)	0.8725 ^2^	-	
Location—chest	19 (3.78%)	5 (8.2%)	0.1663 ^2^	-	
Location—abdomen	1 (0.2%)	0 (0%)	1 ^2^	-	
Location—back	27 (5.38%)	3 (4.92%)	1 ^2^	-	
Location—upper limb	19 (3.78%)	1 (1.64%)	0.7122 ^2^	-	
Location—lower limb	12 (2.39%)	0 (0%)	0.7323 ^2^	-	
Location—limb	31 (6.18%)	1 (1.64%)	0.2373 ^2^		
Location—corpus	47 (9.36%)	8 (13.11%)	0.3599 ^2^		
**Dermoscopic variables**					
White-red/pink structureless areas	424 (84.46%)	39 (63.93%)	0.0006 ^2^	95.37–96.09%	0.865 (0.81–0.92)
Monomorphic vessels ^5^	246 (49%)	31 (50.82%)	0.2895 ^2^	100–97.51%	1 (1–1)
Polymorphic vessels (2 or more vessel types) ^5^	244 (48.61%)	29 (47.54%)	0.2895 ^2^	88.89–92.18%	0.891 (0.853–0.929)
Branched (arborizing) vessels	396 (78.88%)	49 (80.33%)	0.6951 ^2^	97.32–97.87%	0.937 (0.902–0.972)
Superficial fine telangiectasia	294 (58.57%)	37 (60.66%)	0.2921 ^2^	90.91–94.49%	0.887 (0.849–0.926)
Dotted vessels	33 (6.57%)	5 (8.2%)	0.7478 ^2^	82.93–98.76%	0.9 (0.827–0.973)
Coiled vessels	34 (6.77%)	4 (6.56%)	1 ^2^	81.08–98.76%	0.889 (0.808–0.97)
Looped vessels	42 (8.37%)	1 (1.64%)	0.25 ^2^	88.37–99.11 %	0.933 (0.876–0.991)
Helical vessels	0 (0%)	0 (0%)	-	100–100%	1 (1–1)
Curved vessels	7 (1.39%)	0 (0%)	1 ^2^	100–100%	1 (1–1)
Small erosions—mean (median)	1.034 (0)	0.984 (0)	0.984 ^3^	-	0.862 (0.821–0.904)
Ulceration	262 (52.2%)	9 (14.8%)	**<0.0001** ^2^	93.97–96.98%	0.94 (0.911–0.968)
In-focus blue-grey dots	12 (2.39%)	4 (6.56%)	0.1083 ^2^	61.9–98.58%	0.757 (0.595–0.92)
Blue/grey globules	61 (12.15%)	10 (16.39%)	0.5066 ^2^	76.54–96.63%	0.848 (0.781–0.915)
Large blue ovoid nests	43 (8.57%)	6 (9.84%)	0.7944 ^2^	81.82–98.22%	0.89 (0.823–0.957)
Brown dots	57 (11.4%)	20 (32.8%)	**0.0001** ^2^	81.93–97.34%	0.885 (0.828–0.942)
Brown globules	41 (8.2%)	18 (29.5%)	**<0.0001** ^2^	84.62–98.22%	0.907 (0.85–0.964)
Maple-leaf like structures	5 (1%)	3 (4.92%)	**0.0136** ^2^	63.64–99.29%	0.774 (0.56–0.989)
Spoke-wheel-like structures	3 (0.6%)	0 (0%)	1 ^2^	75–99.82%	0.856 (0.578–1)
Concentric structures	5 (1%)	0 (0%)	1 ^2^	55.56–99.29%	0.711 (0.44–0.982)
Starry milia-like cysts	80 (15.94%)	11 (18.03%)	0.0839 ^2^	80.58–96.45%	0.871 (0.816–0.926)
Cloudy milia-like cysts	75 (14.94%)	11 (18.03%)	0.2731 ^2^	84.27–97.51%	0.9 (0.849–0.952)
Cloudy/starry milia-like cysts	57 (11.6%)	16 (26.2%)	**0.0031** ^2^	89.19–98.58%	0.935 (0.89–0.98)
Yellow globules/light yellow globules	10 (1.99%)/36 (7.2%)	1 (1.64%)/10 (16.4%)	0.7202 ^2^/**0.033** ^2^	100–100%/82.35–98.4%	1 (1–1)/0.895 (0.826–0.963)
Multiple aggregated yellow-white globules (MAY-globules)	15 (2.99%)	5 (8.2%)	0.065 ^2^	73.08–98.76%	0.838 (0.72–0.956)
Brown structureless areas	65 (12.95%)	7 (11.48%)	0.8064 ^2^	93.51–99.11%	0.961 (0.928–0.995)

^1^ Cohen Kappa was calculated using irr R package. ^2^ Fisher exact test was used for comparison of binary variables. ^3^ Mann–Whitney U test was used for quantitative data which significantly differed (*p*-value < 0.05) from the normal distribution. Shapiro–Wilk test was used to evaluate whether the distribution is normal. ^4^ Data were missing for 1 BCC patient. ^5^ Data were missing for 12 BCC patients and 1 patient with trichoblastic tumour.

### 3.1. Clinical Variables

In the study, 502 cases of histopathologically confirmed BCC were compared with 61 TT (including 44 TB (72.13%), 10 TE (16.39%) and 7 DTE (11.48%)). Patients in the BCC group were statistically older, compared to those diagnosed with TT (mean age was 71.4 vs. 64.4 years, respectively; *p* = 0.0152; age range 9–94 years for TT, and 12–95 years for BCC). In the BCC group female predominance was observed, in contrast to the latter, however, with no statistical significance (52.0% vs. 44.3%, respectively; *p* = 0.279). BCC presented generally as larger tumours (mean tumour size 11.0 vs. 8.2 mm for the TT group; *p* = 0.001) and more frequently associated with clinically visible ulceration (59.4% vs. 19.7%, respectively; *p* < 0.001). Comparison of lesion morphology (flat vs. elevated) did not show significant differences between the analysed groups. Similarly, no differences were found in the presence of clinically visible pigmentation and anatomical location.

### 3.2. Dermoscopic Variables

Dermoscopically visible ulceration was significantly more common in the BCC group, compared to the TT group (52.2% vs. 14.8%; *p* < 0.0001). Pigmented structures, precisely brown dots and brown globules, were significantly more prevalent in the TT group (32.8% vs. 11.4%; *p* = 0.0001 and 29.5% vs. 8.2%; *p* < 0.0001). Similarly, TT more commonly than BCC showed the presence of cloudy/starry milia-like cysts (26.2% vs. 11.6%, *p* = 0.0031) and yellow globules (16.4% vs. 7.2%; *p* = 0.033).

### 3.3. Diagnostic Algorithm

The binary classification algorithm managed to reach 79.23% AUC (95% CI: 69.69–88.76%) on the randomly selected independent test set. The detailed ROC curve is presented in Figure 5a. While the general performance of the algorithm is good, its clinical utility remains low. When discriminating between malignant and benign tumours, usually a very high sensitivity is preferred, since the cost of false negative is significantly higher than the cost of false positive. Predicted classification scores are depicted in Figure 5b. There is a clear separation between the groups; however, it was not possible to determine a cut-off level, which would assure close to 100% sensitivity, while retaining relatively high specificity. Information from dermoscopic evaluation seems to be insufficient for the reliable and clinically useful discrimination between TT and BCC. The most important features of the model were presence of ulceration, age, maximum size, and the presence of cloudy/starry milia-like cysts. The detailed feature importance is presented in Figure 5c. The overview of the model is presented in Figure 5d.

## 4. Discussion

Epidemiological data regarding trichoblastic tumours remain incoherent. While some studies indicated that TB occurred in younger patients, in others there were no statistically significant differences in the patients’ age [11]. In a study by Ghigliotti et al. [6], the mean age of TB and BCC patients was 62 and 60 years, respectively. The largest reported clinico-histopathological analysis of TT published recently showed that they most commonly occur in the fifth and sixth decade of life [9]. In our study, TT were most commonly diagnosed in the sixth decade of life, and patients with TT were significantly younger compared to those diagnosed with BCC; however, the age range was wide for both groups. While in some studies TT were found to be more common in females, others did not confirm this finding, which is in line with our study [6,9]. Similar to previous studies, the most common location of both BCC and TT was the face, and both groups did not differ in anatomical location [6,9]. Ulceration (both observed clinically and dermoscopically) was significantly more common in the BCC group, which is in line with the previous study by Ghigliotti et al. [6]. The finding of more frequent ulceration in BCC cases may be attributed to the mucinous stroma found in nodular subtype BCC. Mucin is soft and lacks tensile strength. TT, especially DTE, have a more robust tumour stroma, with a lower tendency to ulceration.

In contrast, we did not observe differences in clinically observable pigmentation in either of the analysed groups.

Data concerning dermoscopic presentation of TT are scarce and limited to case reports and small case series [5,6,7]. Ghigliotti et al. [6] analysed the prevalence of 7 predefined dermoscopic criteria (arborizing vessels, blue-grey globules, blue-ovoid nests, ulceration, maple-leaf-like structures, chrysalis, and spoke-wheel structures) of 19 trichoblastic BCC (tBCC) and 19 TB cases and found out that tBCC statistically more commonly displayed blue-grey ovoid nests and blue-grey globules. However, these structures were also observed in a few cases of TB. Notably, the limited number of patients included and patterns analysed could influence the results of the study and fail to identify some subtle differences between both tumour types. In contrast, our findings showed that brown dots and brown globules were significantly more common in the TT group.

Costello et al. [12], in a recently published case series, analysed dermoscopic features of four DT cases which revealed polymorphous vessels (2/4), linear serpentine vessels (2/2), circular white structures (4/4), and shiny white structures (1/4). In a study by Ghigliotti et al. [6], no statistical significance was found between the presence of arborizing vessels in the TB (95%) and BCC (86%) groups, and the presence of chrysalis structures (16 vs. 21%, respectively).

Pitarch et al. [5] described dermoscopy of facial TB in two patients. The first patient’s tumour presented short fine telangiectasia over a whitish background. In the second case, apart from vessels of similar morphology, the authors described white striae and milia-like cysts.

In our study, in both groups, polymorphic vessels were observed in almost half of the analysed cases, with branched (arborizing) vessels and superficial fine telangiectasia most commonly observed. We did not find circular white structures in any of the analysed trichoblastic tumours. Similarly, we did not observe shiny white structures/white striae/chrysalis, as no polarised dermoscopy pictures were available for analysis.

Additionally, we found yellow cloudy/starry milia-like cysts (MLCs) and yellow globules to be more prevalent in the TT group. Previously, Belluci et al. [13], in a study analysing 400 BCC cases, identified the presence of MLCs in 7.75% and yellow globules in 4.2%.

Navarrete-Dechent et al. [14] analysed the prevalence and significance of multiple aggregated yellow-white globules (MAY globules) in BCC. These structures were present in 61 of 291 BCC cases (21.0%) and associated with high-risk BCC diagnosis. In this study, the control group included different skin tumours, including four cases of DTE—in two of them, the authors observed MAY globules as well. Interestingly, in our study, the presence of MAY globules was revealed in only 2.99% of analysed BCC cases and 8.2% of TT.

Study limitations include its retrospective and single-centre character as well as lack of analysis of dermoscopic features visible on polarised dermoscopy. The patients studied represented I-III Fitzpatrick phototype. Thus, the results cannot be easily extrapolated to populations with darker skin phototypes.

Additionally, we did not examine the diagnostic significance of alternative imaging modalities in differential diagnosis of BCC and TT. Previous studies suggested possible utility of in vivo reflectance confocal microscopy (RCM), optical coherence tomography, as well as high-frequency ultrasound examination [6,15,16,17,18,19,20,21,22,23,24,25,26,27,28,29,30].

## 5. Conclusions

Despite differences in frequency of clinical and dermoscopic features between BCC and TT in the studied group, differential diagnosis based on these variables is not reliable based on the developed algorithm, as the overlap of the analysed features was observed in both tumour types. Histopathological examination remains a diagnostic gold standard in differentiation of BCC and TT.

## Data Availability

All data used in the study are available from the corresponding author upon request.
